# Renal function in neonates with twin-twin transfusion syndrome treated with or without fetoscopic laser surgery

**DOI:** 10.1007/s00431-017-2964-2

**Published:** 2017-07-20

**Authors:** Lianne Verbeek, Faiez A. Joemmanbaks, Jacoba M. E. Quak, Ram N. Sukhai, Johanna M. Middeldorp, Dick Oepkes, Enrico Lopriore

**Affiliations:** 1 Division of Neonatology, Leiden University Medical Center, Leiden, The Netherlands; 2 Division of Pediatrics, Leiden University Medical Center, J6-S, Albinusdreef 2, 2333 ZA Leiden, The Netherlands; 3 Division of fetal therapy, Department of Obstetrics, Leiden University Medical Center, Leiden, The Netherlands

**Keywords:** Twin-twin transfusion syndrome, Monochorionic twins, Renal function, Renal insufficiency, Creatinine, Fetoscopic laser surgery

## Abstract

The aim of this study is to investigate the short-term renal function in neonates with twin-twin transfusion syndrome (TTTS), treated with fetoscopic laser surgery (laser group) or conservatively (non-laser group). Creatinine and urea levels and urine output were recorded in the first week after birth. Primary outcome was short-term renal dysfunction, defined as a creatinine level of >100 μmol/L during the first week postpartum. We evaluated 312 twins (laser group, *n* = 274; non-laser group, *n* = 38). Median creatinine and urea levels were lower in the laser group than in the non-laser group (71 versus 82 μmol/L, *p* = 0.002). Short-term renal dysfunction was lower in the laser group compared to the non-laser group (7.2 versus 34.4%, *p* < 0.001). Within the laser group, creatinine levels were significantly higher in the subgroup with incomplete laser surgery compared to twins with successful laser surgery (76 versus 69 μmol/L, *p* = 0.018). No differences were found between donors and recipients except for a higher incidence of oliguria in donors in the non-laser group on day 1.

*Conclusion*: Short-term renal dysfunction occurs less frequently in TTTS twins treated with fetoscopic laser coagulation, particularly after complete surgery, suggesting that laser surgery may have a protective effect on renal function.

**Table:** 

**What is Known:** • *Antenatally, donor twins in TTTS have severe oliguria due to chronic hypovolemia and impaired renal perfusion* • *Postnatally, donor twins may suffer from severe renal complications, particularly in TTTS twins treated conservatively*.
**What is New:** • *The incidence of short-term renal failure in TTTS twins treated with complete laser surgery is low*. • *After incomplete laser surgery, the incidence of short-term renal dysfunction is increased*.

## Introduction

Twin-twin transfusion syndrome (TTTS) is a serious complication that occurs in 10–15% of monochorionic twin pregnancies and results from unbalanced inter-twin blood transfusion through placental vascular anastomoses [1]. TTTS is detected by prenatal ultrasound and is characterized by the presence of polyhydramnios in the recipient twin due to hypervolemia and polyuria, and oligohydramnios in the donor due to hypovolemia, renal hypoperfusion, and oliguria. TTTS can be treated antenatally with serial amnioreduction, but the optimal treatment is coagulation of the vascular anastomoses with fetoscopic laser surgery [2]. Laser surgery is associated with a significant reduction in perinatal morbidity and mortality [3]. As a result of the increase in perinatal survival, attention is now shifting toward short-term and long-term morbidity in survivors. Several small studies in TTTS not treated with laser surgery reported various renal complications in donor twins after birth, caused by impaired renal perfusion, including histological renal changes such as hypovascularisation and microangiopathy [4–8]. Whether postnatal renal dysfunction also occurs in twins affected by TTTS treated with laser surgery is not well known, as only few small studies have been published to date [9, 10].

The aim of this retrospective study is to investigate the short-term postnatal renal function in a large cohort of TTTS twins treated with laser surgery (laser group), compared to a group of TTTS twins treated conservatively with either serial amnioreduction or expectant management (non-laser group) and evaluate potential risk factors such as incomplete laser surgery and donor status.

## Methods

Data of all consecutive monochorionic twin pairs with TTTS, born at the Leiden University Medical Center (The Netherlands) between July 2009 and June 2016, were collected. The Leiden University Medical Center is a tertiary care center and serves as the national referral center for monochorionic twin pregnancies with TTTS.

TTTS was diagnosed using antenatal ultrasound, according to the Eurofoetus criteria [2]. Pregnancies with intrauterine death of one or both fetuses or twins with congenital anomalies of the kidney and urinary tract were excluded.

Laser surgery was performed up to 26 weeks of gestation in all cases with stage 2 TTTS or higher and in cases with stage 1 associated with symptomatic polyhydramnios. All other cases were treated conservatively with either serial amnioreduction or expectant management.

Outcome of twins treated with laser surgery (laser group) was compared to twins treated conservatively (non-laser group) and between donors and recipients. In the laser group, outcome was compared between twins with successful laser surgery (complete laser group) and the twins with either recurrent or reversal of TTTS or post-laser twin anemia-polycythemia sequence (TAPS) (incomplete laser group). The following perinatal variables were recorded: gender, mode of delivery, gestational age at birth, birth weight, and birth weight difference between both twins. Birth weight difference was calculated as follows: ((birth weight larger twin − birth weight smaller twin)/birth weight larger twin) × 100.

At birth, blood pressure, heart rate, and hemoglobin levels were routinely measured in all twins as standard of care. In addition, creatinine and urea levels were routinely measured in the first week after birth. Values measured in the first 2 days after birth were excluded to reduce the influence of maternal creatinine and urea. The highest value was included if more than one value was measured. Urine output (in ml/kg/h) and the presence of oliguria (urine output <1 ml/kg/h) during the first 3 days after birth were recorded.

The following neonatal data were collected as well: hypotension, respiratory distress syndrome (RDS), necrotizing enterocolitis (NEC), patent ductus arteriosus, sepsis, fetal distress, cerebral injury, and neonatal mortality. Hypotension was defined as low blood pressure for which inotropic medication was given. Low blood pressure is defined as a mean blood pressure lower than the gestational age. RDS was defined as respiratory failure requiring respiratory support with surfactant therapy. We recorded NEC in the case of ≥stage 2, according to the Bell’s criteria. Cerebral injury was defined as any of the following: cystic periventricular leukomalacia or intraventricular hemorrhage grades 3–4. Sepsis was defined as blood culture-proven sepsis or clinical sepsis (defined as a clinically ill neonate with elevated infection parameters, requiring antibiotic treatment, but without a positive blood culture). Fetal distress was defined as the presence of at least three if the following criteria: decelerative cardiotocogram, arterial umbilical cord pH < 7.10, 5-min Apgar score < 5, spontaneous breathing > 5 min after birth, or multi-organ failure.

The primary outcome in this study was short-term renal dysfunction, defined as a creatinine level of >100 μmol/L in the first week of life. We defined severe renal dysfunction as a creatinine level of >150 μmol/L. Secondary outcomes were urine output and oliguria in the first 3 days of life.

The local Medical Ethics Committee provided a statement of no objection for obtaining and publishing the anonymized data.

### Statistics

Data were reported as median and interquartile range (IQR). Results of continuous variables between two groups were analyzed using a Mann-Whitney *U* test. In order to compare donors and recipients, we used the paired sample *t* test for continuous variables and the Mc Nemar test for categorical variables. Because of the limited sample size in the non-laser group, a Fisher-Exact test was used for nominal variables. Two-sided tests were used for statistical analyses, and a *p* value <0.05 was considered statistically significant. All analyses were performed using SPSS version 23 (SPSS, Inc., Chicago, Ill, USA).

## Results

During the study period, 312 twins fulfilled the inclusion criteria, of which 274 twins were treated with fetoscopic laser surgery (laser group), and 38 twins were treated conservatively (non-laser group) and managed either expectantly (*n* = 23) or with serial amniodrainage (*n* = 15). The baseline characteristics of the two studied groups are summarized in Table 1. The gestational age at diagnosis of TTTS was 19 (17–22) weeks in the laser group and 28 (26–29) weeks in the non-laser group. TTTS staging at diagnosis was two (1–3) in the laser group and one (1–3) in the non-laser group. In the laser group, the median gestational age at laser surgery was 19 (17–22) weeks.

**Table 1:** Baseline characteristics in twins affected by TTTS treated with laser surgery (laser group) and treated conservatively (non-laser group)

	Laser group (*n* ^a^ = 274)	Non-laser group (*n* ^a^ = 38)
TTTS stage at diagnosis^b^	2 (1–3)^c^	1 (1–3)^d^
TTTS stage 1—*n* (%)	72 (26.3%)^c^	16 (42.1%)^d^
Gestational age at diagnosis—week^b^	19 (17–22)^e^	28 (26–29)^d^
Gestational age at birth—week^b^	32 (30–34)	30 (29–34)
Cesarean section—no (%)	114 (41.6%)	30 (78.9%)
Female—no (%)	134 (48.9%)	14 (36.8%)
Birth weight—g^b^	1652 (1257–2046)	1548 (1039–2205)
Birth weight difference—%^b^	10.5 (5.2–20.6)	9.8 (8.1–21.7)

^a^Refers to the number of neonates

^b^Value given as median (IQR)

^c^4.4% (12) missing

^d^26% (10) missing

^e^3.6% (10) missing

Results of renal function in the laser group and non-laser group are shown in Table 2. Creatinine and urea levels were significantly lower in the laser group compared to the non-laser group, respectively 71 versus 82 μmol/L (*p* = 0.002) and 5.2 versus 7.6 mmol/L (*p* < 0.001). Short-term renal dysfunction (creatinine level of >100 μmol/L) occurred less often in the laser group (7.1%) compared to the non-laser group (37.9%) (*p* < 0.001).

**Table 2:** Renal function in the laser group and non-laser group and between the subgroup with complete laser and incomplete laser treatment

				Laser group		
	Laser group (*n* ^a^ = 274)	Non-laser group (*n* ^a^ = 38)	*P* value	Complete laser (*n* = 228)	Incomplete laser (*n* = 46)	*P* value
Creatinine week 1—μmol/L^b^	71 (61–81)^c^	82 (69–148)^d^	0.002	69 (59–80)^e^	76 (66–87)^f^	0.018
Creatinine >100 μmol/L—*n* (%)	12 (7.1%)^c^	11 (37.9%)^d^	<0.001	6 (5%)^e^	6 (15%)^f^	0.073
Urea week 1—mmol/L^b^	5.2 (3.6–7.1)^g^	7.6 (5.8–11.5)^d^	<0.001	5.1 (3.6–7.1)^h^	6.2 (4.4–7.2)^f^	0.178
UO day 1 <1 ml/kg/h—*n* (%)	69 (40.6%)^i^	14 (60.9%)^j^	0.075	55 (42%)^k^	14 (37%)^l^	0.708
UO day 2 <1 ml/kg/h—*n* (%)	8 (4.8%)^m^	9 (36.0%)^n^	<0.001	5 (4%)^e^	3 (8%)^l^	0.387
UO day 3 <1 ml/kg/h—*n* (%)	4 (2.6%)^o^	2 (8.3%)^p^	0.192	2 (2%)^q^	2 (5%)^r^	0.252
MBP at birth—mmHg^b^	37 (33–43)^s^	39 (32–52)^t^	0.349	37 (33–42)^u^	39 (33–45)	0.247
Heartrate at birth—bpm^b^	154 (143–166)^v^	146 (131–158)^t^	0.004	154 (142–166)^w^	156 (149–164)	0.298

*UO* urine output, *MBP* mean blood pressure, *bpm* beats per minute

^a^Refers to the number of neonates

^b^Value given as median (IQR)

^c^38.6% (106) missing

^d^23.7% (9) missing

^e^0.8% (1) missing

^f^10.8% (5) missing

^g^1.8% (5) missing

^h^43.9% (100) missing

^i^38.0% (104) missing

^j^39.5% (15) missing

^k^42.1% (96) missing

^l^17.4% (8) missing

^m^39.8% (109) missing

^n^34.2% (13) missing

^o^48.5% (133) missing

^p^36.8% (14) missing

^q^50% (114) missing

^r^19.6% (9) missing

^s^19.3% (53) missing

^t^13.2% (5) missing

^u^23.2% (53) missing

^v^13.1% (36) missing

^w^15.8% (36) missing

The incidence of oliguria was significantly lower in the laser group, compared to the non-laser group, but only on day 2.

Neonatal mortality and several perinatal and neonatal morbidities occurred less frequently in the laser group compared to the non-laser group, including hypotension and fetal distress, as shown in Table 3. Renal function was only in assessed in two of the four neonates with a fetal distress, since two neonates died within 24 h after birth.

**Table 3:** Clinical outcome in TTTS twins in the laser group and the non-laser group

	Laser group (*n* ^a^ = 274)	Non-laser group (*n* ^a^ = 38)	*P* value
Hypotension—*n* (%)	12 (4.5%)^b^	8 (21.1%)	0.001
Cerebral injury—*n* (%)	8 (2.9%)	1 (2.7%)^c^	1.000
Necrotizing enterocolitis—*n* (%)	3 (1.1%)^g^	1 (2.6%)	0.408
Patent ductus arteriosus—*n* (%)	37 (13.5%)	10 (26.3%)	0.051
Sepsis—*n* (%)	25 (9.1%)	5 (13.5%)^c^	0.377
Fetal distress—*n* (%)	1 (0.4%)^d^	4 (11.1%)^e^	0.001
Mortality—*n* (%)	8 (2.9%)	5 (13.2%)	0.013

^a^Refers to the number of neonates

^b^2.2% (6) missing

^c^2.6% (1) missing

^d^0.4% (1) missing

^e^5.3% (2) missing

Within the laser group, 228 had a successful laser surgery (complete laser group) and 46 twin pairs had incomplete laser surgery (post-laser TAPS, *n* = 36; recurrent or reversal TTTS, *n* = 10), as shown in Table 2. The median creatinine level in the complete laser subgroup was significantly lower (69 μmol/L) compared to median creatinine level in the incomplete laser group (76 μmol/L) (*p* = 0.018). The number of neonates with a creatinine level of >100 μmol/L and the incidence of oliguria was similar in both subgroups.

In the non-laser group, donor twins had more often oliguria on day 1 (92.3 versus 20.0%, *p* = 0.001) and a lower mean blood pressure (34 versus 47 mmHg, *p* = 0.031) compared to their recipient co-twin. We found no other differences between donors and recipients regarding other outcomes, including short-term renal dysfunction, creatinine levels, and urea levels (data not shown).

A creatinine level of >150 μmol/L was detected in 10 neonates and occurred less often in the laser group than in the non-laser group, respectively 1.1 (3/274) versus 18.4% (7/38) (*p* < 0.001) and more often in donors (7/10) than recipients (3/10). In the laser group, severely elevated creatinine levels were detected in 4.4% (2/46) in the incomplete laser group and in 0.4% (1/228) in the complete laser group. Neonatal mortality occurred in three of the ten neonates and was due to multi-organ failure (*n* = 2) and respiratory failure including recurrent bilateral tension pneumothorax and respiratory distress syndrome (*n* = 1). The highest creatinine level (347 μmol/L) was detected in a donor twin in the non-laser group and was thought to be due to acute tubular necrosis after severe chronic renal hypoperfusion. Creatinine levels and renal function in this donor twin normalized within several weeks after hyperhydration. Renal ultrasound showed nephrocalcinosis. Urine calcium/creatinine ratio was not elevated, and on the follow-up ultrasound at age 2, the nephrocalcinosis was barely visible.

## Discussion

This is the first study on short-term renal function in a large cohort of TTTS twins treated with or without laser surgery, assessing also the impact of incomplete laser treatment. We found that the incidence of short-term renal failure in TTTS twins treated with laser surgery is low. In contrast, the incidence of renal failure and oliguria is increased in TTTS treated conservatively or after incomplete laser surgery. Our findings therefore suggest that laser surgery, when complete, may protect fetuses and neonates against renal injury. Although donor twins suffer from severe oliguria and oligohydramnios, these symptoms resolve after laser surgery (reflected by the reoccurrence of amniotic fluid in the donor’s sac) antenatally and detection of normal renal function in the vast majority of survivors after birth. However, long-term evaluation of renal function in large TTTS cohorts is required to assess if the protective effect is also present after the initial neonatal period.

A few small studies reported on the short-term and long-term renal function in TTTS survivors after fetoscopic laser surgery. Halvorsen et al. found a slightly higher incidence of renal failure in a cohort of TTTS twins after laser surgery 10% (9/87) compared to our cohort, but criteria for the renal dysfunction were not provided [11]. Lenclen et al. also reported a similar risk of renal failure in TTTS twins after laser surgery (7.1%, 7/98), but again, the definition of renal failure was not specified, preventing comparisons with our findings. In addition, only preterm neonates delivered before 30 weeks of gestation were included in this study [10].

Although our data suggest that the vast majority of TTTS twins after laser surgery do not have short-term renal dysfunction, a few neonates still had severely increased creatinine levels. This may partly be explained by the incomplete laser surgery and persistence of inter-twin blood transfusion. Whether the risk of renal dysfunction is also low on the long-term is not well known, as this was evaluated only in one small study from Beck et al. in 18 TTTS twins treated with laser surgery. They found no evidence of renal failure at a median age of 3 years; however, the sample size was too small to reach firm conclusions [9]. Accurate renal follow-up should take much longer. Due to the large reserve capacity of the kidney, serum creatinine could be normal for a long time. When children grow older and particularly during the growth spurt, renal insufficiency might become evident [12].

In accordance with our findings, most studies in TTTS twins not treated with laser report an increased risk of short-term renal dysfunction, in particular in donor twins. In a pathology study evaluating the renal anatomy in 25 TTTS twin pairs after autopsy, De Paepe et al. found a loss of proximal convoluted tubules in 48% of donors and 48% of recipients. Although the glomerular density was higher in donor kidneys, the number of glomerular generations was similar in donors and recipients. The kidney weight of recipients was almost twice as large of that of donor twins [5]. By comparison, Oberg et al. reported in 8/9 donors varying degrees of tubular deficiency [13], and Barr et al. reported varying degrees of tubular deficiency in 11/21 donor kidneys (but in none of the 17 recipient twins) [8]. In a study by Chiang et al. in 22 neonates with TTTS, nine (41%) had acute renal failure (defined as serum creatinine level above 1.5 mg/dL (133 μmol/L) regardless of urine amount), of which the vast majority (8/9) were donors [4]. In a study performed by our research group in 56 TTTS neonates treated conservatively, renal failure detected in two (4%) neonates, both donors, of which one died of terminal renal failure. Definition of renal failure was not described [14]. Cincotta et al. found an increased risk of renal failure (defined as urine output <1 ml/kg/h during the first 3 days of life and high creatinine levels) in a small group of TTTS twin pairs (*n* = 17) treated conservatively compared to uncomplicated monochorionic twins (48 versus 15%, *p* = 0.005), but no differences between donors and recipients were found [15]. Lastly, Lenclen et al. also found an increased incidence of renal failure in TTTS twins treated with amniodrainage (20.0%, 6/30). However, no differences between donors and recipients were found.

In these studies, renal dysfunction which was detected mainly in donors is thought to result mainly from chronic poor renal perfusion and hypovolemia, in association of chronic hypoxia and anemia [4, 10, 14, 15].

In our study, we also found a high risk of oliguria in donors (>90%) in the non-laser group and a lower blood pressure at birth, both probably reflecting the hypovolemic state of these neonates at birth.

The ex-recipient, after complete laser surgery, has no renal dysfunction due to good recovery in the antenatal period. In case of incomplete laser surgery, the risk of renal dysfunction is increased in both ex-donors and ex-recipients due to persistent hemodynamic instability in utero. However, due to small sample size and lack of power, the inter-twin differences in this subgroup were not statistically significant.

The main limitation of this study is its retrospective design and a selection bias between the conservative and the laser group. The two groups differed in term of presenting characteristics. The non-laser group was small and heterogeneous, as it contained a mixture of TTTS cases with lower TTTS stages and/or presentation at a later gestational age. Since TTTS in the non-laser group was less severe, the incidence of renal dysfunction in this subgroup was probably underestimated. In contrast, the laser group in this study is very large and homogeneous, limiting the risk of bias and supporting the reliability of our findings. However, we included only TTTS cases with double survivors at birth (to compare the outcome within twin pairs); therefore, renal function in survivors after single fetal demise requires further investigation. As recently shown, single fetal demise in TTTS twins not treated with laser can lead to severe renal ischemia and terminal renal failure due to acute exsanguination through the vascular anastomoses [16]. Since dichorionization of the placenta after complete laser surgery prevents acute exsanguination, this selection bias with double survivors may again have led to an underestimation of the risk of renal failure in the non-laser group. Another limitation is that other factors than TTTS can influence renal function, such as being born at a younger gestational age or hypotension. Unfortunately, due to the small cohorts of the subgroups, the possibilities to perform subgroup analysis and corrections for confounders are limited and unreliable. Larger cohorts are needed to address these pertinent questions. Finally, the assessment of short-term renal function in this study was based only on routine measurements at our neonatal nursery including urine production, creatinine, and urea levels during the first week. More detailed information on renal function would require different assessments and a different study design.

In conclusion, our findings show that the risk of short-term renal dysfunction in TTTS treated with laser surgery is low, suggesting a protective effect of laser coagulation despite the presence of severe oliguria and oligohydramnios in donor twins during fetal life. Therefore, routine evaluation of renal function after complete laser surgery in all survivors does not seem warranted. In contrast, after incomplete laser surgery or in TTTS treated conservatively, the risk of renal dysfunction is increased, and postnatal renal evaluation should be recommended. Future prospective research should focus on long-term renal outcome in TTTS treated with and without laser surgery to assess if the findings in the neonatal period persist through childhood and adulthood.

## Abbreviations

TAPS: twin anemia-polycythemia sequence
TTTS: twin-twin transfusion syndrome
